# Road Crashes in Adults with Attention Deficit Hyperactivity Disorder and Risky Driving Behavior

**Published:** 2020-04

**Authors:** Hajar Sadeghi, Yazdan Shabani, Abdolghader Pakniyat, Kiandokht Karimian, Mehdi Harorani, Yazdan Naderi Rajeh

**Affiliations:** 1 Student Research Committee, University of Social Welfare and Rehabilitation Sciences, Tehran, Iran.; 2 Student Research Committee, Arak University of Medical Sciences, Arak, Iran.; 3 School of Medicine, Kurdistan University of Medical Sciences, Sanandaj, Iran.; 4 Emergency Medicine Department, Arak University of Medical Sciences, Arak, Iran.; 5 School of Nursing and Midwifery, Arak University of Medical Sciences, Arak, Iran.; 6 Psychiatry and Behavioral Sciences Research Center, Addiction Institute, Mazandaran University of Medical Sciences, Sari, Iran.

**Keywords:** *Attention-Deficit Hyperactivity Disorder (ADHD)*, *Car Accident*, *Risky Behavior*

## Abstract

**Objective:** Attention deficit hyperactivity disorder (ADHD) is one of the most common problems in adolescents. Risky behaviors in patients with ADHD are due to impaired impulse control resulting from problems with inhibition of proponent responses, controlling interference, and stopping ongoing responses after feedback on errors. The present study investigated the relationship between ADHD and risky driving behavior and the likelihood of car accident in Arak, Iran, in 2015-16.

**Method** **:** This case-control study was conducted in the Emergency Department of Vali-Asr hospital in Arak (Iran) on drivers who met the inclusion criteria. The data gathering tools included the Demographic Questionnaire, Manchester Driving Behavior Questionnaire (MDBQ), and Wender Utah Rating Scale (WURS). Statistical analyses were performed using SPSS version 20 software.

**Results: **The mean of ADHD (±SD) was higher among cases (81.64 [26.78]) than in controls (64 [24.28], P = 0.000). The mean of risky driving behaviors (±SD) was higher among cases (66.41[26.78]) than in controls (36.79 [25.42]). There was a significant relationship between ADHD, risky behavior, lapse errors, slips, deliberate ‎violation, and unintentional violation and car accident (P = 0.000).

**Conclusion: **This study showed that ADHD increases the risk of road crashes and motor vehicle injuries. These drivers tend to drive at unauthorized speed, have less control over the vehicle, drive more carelessly, and are more likely to have an accident.

ADHD is known as a risk factor for traffic crashes. In the past, research has shown a relationship between driving and individuals with attention-deficit hyperactivity disorder (ADHD) (1). This common psychiatric disorder often persists throughout the lifespan, and according to the diagnostic criteria, ADHD is characterized by such symptoms as hyperactivity, attention deficit, and impulsivity (2-4). Different studies have shown that 40% to 70% of people who had ADHD in childhood experience symptoms of this disease until adulthood (2). Complications associated with ADHD include suicide, addiction, increased criminality, injury, and risky behaviors (5-7). In addition, sleep disturbance is common in these patients, which affects their daily behaviors, as well as the related emotional liability and a high variation in erroneous behavior (8, 9). The unintended consequences of this disease in adults include poor interpersonal relationships, events and traumas due to impulsivity, family conflicts, educational problems, problems in marital and parental relationships, alcohol and drug abuse, antisocial behaviors, and early sexual relationships. Furthermore, their impulsivity may result in increased car and other types of accidents (2). These adults tend to drive at unauthorized speed and have less control over the vehicle (10, 11). 

Risky behaviors in patients with ADHD are due to impaired impulse control because of problems with inhibition of proponent responses, controlling interference, and stopping ongoing responses after feedback on errors (5-7). Almost 95% of accidents have occurred due to risky driving behaviors (12). Risky driving behaviors are as follow: failing to yield right-of-way, blocking intersections, weaving in/out of traffic, failing to stop for pedestrians, making threats or communicate insults through words and/or gestures (obscene gestures or profanity), tailgating, chasing other vehicles, forcing someone to give way, improper passing and lane changing, using 2 lanes, passing on the shoulder, speeding, failing to use signals, changing lanes without signaling, overtaking on the right side, blocking other vehicles, changing lanes, or merging into traffic, making a sudden intentional brakes, cutting in front of other drivers, sustained horn-honking or flashing headlights, preventing others from passing, driving through a yellow light that is turning red, changing speed erratically, taking up more than 1 parking space, pulling into a parking space for which someone else has been waiting, and double parking(13). Some driving behaviors that are beyond the scope of the law, such as breaking the speed limit, illegal turns, improper passing and lane-usage, tailgating, right-of-way violations, and control signal violations that place drivers at risk for morbidity and mortality are known as risky driving behaviors. These behaviors can be either deliberate such as violations, or be due to unintentional errors and distractions (13, 14). about 1.2 million annual mortalities in the world are related to road traffic injuries. Road traffic accidents are considered to be the second highest cause of mortality in Iran (2). Injuries due to traffic accidents will become the fifth leading cause of death by 2030 if preventive interventions are not implemented quickly (WHO) (15). The psychiatric and psychological aspects of injury are an important field of study in accident-ology (16-20). According to the undeniable role of the psychiatric and psychological aspect of road crashes and crash injuries and a high number of motor vehicle accident in Iran, it is necessary to investigate psychiatric and psychological aspect of road crashes and crash injuries. Previous studies have found that ADHD symptoms and difficulty regulating emotions play a role in high-risk driving behaviors. However, the mechanisms underlying these factors have not been studied to date, and it has not been elucidated whether ADHD symptoms have a direct role in driving behavior. This is important because it can address the role of interacting factors. This study clarifies some key issues in the prevention of traffic accidents and is effective in informing traffic experts to identify the risk factors of a traffic accident and to identify drivers at risk. The relationship between the variables in this study was not straightforward. ADHD and risky driving behavior are known as risk factors for road crashes. In the present article, we provided an overview of these studies and aimed to find whether individuals with ADHD have shown increased levels of unsafe driving behavior. The answers to these questions are of high relevance for patients, health specialists, and society alike.

## Materials and Methods

We performed a case-control study to investigate the relationship between adults’ attention-deficit hyperactivity disorder, risky driving behaviors, and motor vehicles injuries. The study was conducted in the Emergency Department of the Vali-Asr hospital in Arak (Iran), which serves urban and rural populations of West of Iran. Patients were recruited from October 2015 to March 2016. Sample size was calculated based on previous studies (2). The patient group consisted of 100 drivers who were involved in a car crash and referred to the Emergency Department of the Vali-Asr hospital in Arak, and the control group consisted of 102 drivers without a history of car crash. Data were collected by trained research interviewers through direct interviews using a standardized questionnaire. Doctor of clinical psychology conducted semi-structured interviews to diagnose any mental disorder in the drivers. The diagnosis of ADHD was based on an interview with a clinical psychologist and a questionnaire interpretation. 

The inclusion criteria in the case group were as follow: admission to Vali-Asr hospital in Arak, age older than 18 years, a history of accident based on self-report, being injured due to a motor vehicle traffic accident, and consenting to participate in the study. The inclusion criteria in the control group were as follow: not having a history of accident based on self-report, age older than 18 years, and consenting to participate in the study. The exclusion criteria in the case group were as follow: very severe injury preventing interview during the hospital stay, and having a history of cognitive disorder based on self-report that would render the interview unreliable. 

In this study, the Persian version of Manchester Driving Behavior Questionnaire was used. It is a valid and reliable questionnaire with 50 items, each having a 6-point Likert scaled answer from 0 to 5. This scale has been translated into Persian and validated by Oreyzi et al. The results of the reliability analysis of the Oreyzi et al (2010) study showed that all 4 factors of this scale have high internal consistency coefficients (slip: 0.77, errors: 0.81, deliberate violations: 0.86, unintentional violations: 0.65) and that the Driving Behavior Questionnaire is a reliable and valuable tool which can be used in driving behavior research (21). Questions have 2 ‎different aspects: One is about the kind of behavior, and another relates to amount of ‎risk posed to other drivers. Abnormal driving behaviors (risky driving behavior) are as follow: Lapse errors, slips, deliberate ‎violation, and unintentional violation. The degree of ‎risk posed to other drivers may be categorized as follows: behaviors that pose no risk to others, and just give a feeling of comfort (low-risk ‎probability), behaviors that are likely to put others at risk (moderate -risk probability), ‎ and those that certainly put others at risk (high-risk probability) ‎ (22). 

The Wender Utah Rating Scale (WURS) is a self-report questionnaire used to retrospectively diagnose the occurrence of attention-deficit hyperactivity disorder in childhood in the age group 18 years and above and confirms the occurrence of attention deficiency hyperactivity disorder in childhood (23). It seems this measure separates adults with ADHD from depressed and abnormal adults (24). Wender Utah Rating Scale can be divided into 6 factors: dysthymia, oppositional defiant disorder, school work problems, conduct disorder, anxiety disorder, and ADHD. This scale can be used to diagnose ADHD in adulthood and has 25 items related to ADHD diagnosis. A total of 60 questions are about remembering childhood behaviors. Ratings are made on a 5-point scale, ranging from 0 to 4 (24-26). This measurement has been applied extensively in various studies, and its reliability and reactivity has been reported to be favorable (25). Also, the sensitivity and specificity of this tool have been reported to be 85% and 76%, respectively. Cronbach’s alpha has been reported to be 0.91 and correlation coefficient has been reported to be 0.85 (24). The ethics committee of Arak Medical University approved this study (IR.ARAKMU.REC.1394.188). 

***Statistical Analysis***

In this study, descriptive statistics were used to determine mean and standard deviation. Descriptive analysis and bivariate tests of association were used prior to multivariate binomial regression analysis to estimate the odds ratios of ADHD and risky riding behavior scales. Also,

 P < 0.05 was considered to be statistically significant. Scores on each factor of the MDBQ (Lapse errors, slips, deliberate ‎violation, and unintentional violation) and ADHD were compared between case and control groups using independent samples t tests. Statistical analyses were out using SPSS version 20 software. 

## Results

Of the 202 participants enrolled in the study, 102 (50.49%) drivers involved in a car accident, with a mean (±SD) age of 35.67 (12.41) were compared with 100 (49.51%) drivers without car accident, with a mean (±SD) age of 32.89 (12.28). All participants were men and their mean age was 34.26±12.4 years. Of the participants, 67.3% were married and 58.4% were the head of their own family. Table 1 shows the participants’ characteristics. ADHD mean (±SD) was higher among cases (81.64 [26.78]) than controls (64 [24.28]), P = 0.000; (t test (P value) = 4.81(0.000). Mean (±SD) of slips among cases and controls were 31.30 (12.89) and 14.89 (10.99), respectively (t test (P value) = 9.56 (0.000)). Cases had higher mean (±SD) of deliberate ‎violation than controls (19.71[9.07] vs 14.31[10.52], respectively (t test (P value) =3.80(0.000)). Unintentional violations mean (±SD) among cases and controls was 5.39 (1.95) and 2.4 (2.25), respectively, [t test (P value) = 9.92(0.000)]. Cases had higher mean (±SD) lapse errors than controls: 10.7(5.26) vs 6.23 (4.93), respectively [t test (P value) = 6.18(0.000)]. Generally, risky driving behavior mean (±SD) was higher among cases (66.41[26.78]) than controls (36.79 [25.42]) (t test (P value) = 7.69(0.000)) (Table 2). The following factors were significantly associated with an increased risk of road crashes and motor vehicle injuries: ADHD (OR = 0.973; 95% CI [1.09-0.824]), slips (OR = 1.628; 95% CI [1.149–2.308]), deliberate ‎violation (OR = 2.229; 95% CI [1.580–3.146]), unintentional violations (OR = 0.449; 95% CI [0.318–0.633]), lapse errors (OR = 1.650; 95% CI [1.172-2.322]), and risky driving behavior (OR = 0.545; 95% CI [.400 -.745]) (Table 3).

**Table 1:** Distribution of Sociodemographic Data of the Case and Control Groups

		**Case**		**Control**	
		**N**	**%**	**n**	**%**
Age	<20	12(11.8)		3 (3)	
	20-40	67(65.7)		67 (67)	
	40-60	18(17.6)		28 (28)	
	>60	5(4.9)		2(2)	
Marriage	Married	68 (66)		68(68.7)	
	Single	33 (32)		29(29.3)	
	Divorced	1(1)		2(2)	
	Wife died	1(1)		0(0)	
Job	Worker	11(10.9)		10(10.3)	
	Employee	17(16.8)		19(19.6)	
	Free job	62(61.4)		66(68)	
	Student	6(5.9)		2(2.1)	
	Unemployed	4 (4)		0(0)	
Education	Illiterate	6(5.9)		0(0)	
	Elementary	14(13.7)		3(3.1)	
	Cycles	39(38.2)		25(25.8)	
	Diploma	15(14.7)		39(30.9)	
	Academic	28(27.5)		39(40.2)	
Income level	Very good	10(9.9)		11(11.2)	
	Good	45(44.6)		55(56.1)	
	Average	30(29.7)		23(23.5)	
	Weak	2 (2)		5(5.1)	
	Very weak	14(13.9)		4(4.1)	
Head of family	Father	35(34.7)		30(30.3)	
	Mother	1(1)		4 (4)	
	Me	60(59.4)		58(58.6)	
	Husband	2 (2)		4 (4)	
	Children	3 (3)		3(3)	
Living with	Lonely	1 (1)		4(4.1)	
	Parents	39(38.2)		25(25.5)	
	Family	62(60.8)		69(70.4)	
Medical history	Yes	19(18.4)		4 (4)	
	No	84(81.6)		95 (95)	

**Table 2:** Mean (±SD) of ADHD and Risky Behavior Subscales in the Case and Control Groups

		**Case**	**Control**
ADHD	Mean	81.64	64.00
	SD	26.78	24.28
	T test (P value)	4.81(0.000)	
Risky behavior	Mean	66.41	36.79
	SD	26.78	25.42
	T test (P value)	(0.000) 7.69	
Slips	Mean	31.30	14.89
	SD	12.89	10.99
	T test (P value)	(0.000) 9.56	
Deliberate ‎violation	Mean	19.71	14.31
	SD	9.07	10.52
	T test (P value)	(0.000) 3.80	
Unintentional violations	Mean	5.39	2.4
	SD	1.95	2.25
	T test (P value)	(0.000) 9.92	
lapse errors	Mean	10.7	6.23
	SD	5.26	4.93
	T test (P value)	(0.000) 6.18	

**Table 3:** Regression Coefficients in Logistic Regression Model for ADHD and Risky Driving Behaviors

	B	S.E.	Wals	Df	Sig	Exp(B)	Lower	upper
							Confidence intervals( CI) for EXP(B)= 95%	
ADHD	-0.027	0.00 6	17.252	1	0.000	0.973	1.09	0.824
Risky behavior	-0.606	0.15 9	14.569	1	0.000	0.545	0.400	0.745
Slips	0.488	0.17 8	7.503	1	0.006	1.628	1.149	2.308
Deliberate ‎violati on	0.802	0.17 6	20.808	1	0.000	2.229	1.580	3.146
Unintentional violations	-0.802	0.17 6	20.808	1	0.000	0.449	0.318	0.633
lapse errors	0.501	0.17 4	8.247	1	0.004	1.650	1.172	2.322
Constant	2.484	0.47 2	27.667	1	0.000	11.989	***	■■■■■■■■■■

## Discussion

The present study aimed to investigate the relationship between road Crashes in adults with ADHD and risky driving behavior. The findings of this study showed that the average of ADHD and high-risk driving behaviors were higher in the case group than in the control group. As predicted and in line with previous research, this study showed that ADHD and risky driving behaviors increase the risk of road crashes and motor vehicle injuries. Inattention, careless and imagination that there is no risk cause their accidents. In this study, most of the participants in both case and control groups were 20-40 years old, married, self-employed, heads of their family, and living with their family. Based on the results of Alavi et al (2017) study, older age reduces the occurrence of accidents (positive effect), which means that the odds of accident occurrence is reduced by 0.01-fold per 1 year age increase (27). The results revealed that education did not increase or reduce the chance of risky behavior. Despite these results, some studies found a relationship between education and driving safely. Habibi, Haghi and Maracy (2014) reported that educational level is an effective factor in driving behavior. Drivers with adequate education have more logical perception of environmental conditions and dangerous agents and pay more attention to traffic boards and blocks (28).

Most of the participants had cycles, good income, and no medical history, respectively (Table 1). Some studies reported that personality can affect drivers’ behaviors and accident involvement among drivers aged 20 to 50 (12). Alavi et al (2017) study revealed that age was associated with a decrease in odds of traffic infringements, meaning that young drivers exhibit more risky driving behaviors than the older (27). Yang et al study revealed that personality traits play an important role in predicting risky driving behaviors of the Chinses. In addition, Chinese drivers’ personality characteristics were also associated with accident involvement (12). Study Results by Alavi et al (2017) revealed that there were differences between means of the Two Groups of drivers, with violation and without violation groups in driving behavior, slips, and laps error. Therefore, understanding of a greater behavioral and cognitive control may help drivers who represent the category at the highest odds of traffic violations and accidents (27). 

The mean of ADHD (±SD) in case and control groups was 81.64 (26.78) and 64(24.28), respectively, which is similar to El Farouki study. El Farouki et al proposed that distraction induced by an external event, distraction induced by an internal thought, and ADHD are associated with responsibility for crashes. Their study showed a higher risk of being responsible for road crashes when exposed to both external distraction and ADHD (15). In this study, the mean of risky driving behaviors was higher in the case group than in the control group, which is similar to Sadeghi-Bazargani study. This case-control study showed that ADHD and riding behavior scores affect the likelihood of motorcycle traffic injuries among motorcycle riders independent of other injury indicators (20). In Alavi et al (2017) study, it was observed that the underlying factors of traffic accidents were human, cognitive, and behavioral factors, such as mental illness, personality, or the age of drivers (29).

The present study showed that risky driving behavior, including lapse errors, slips, deliberate ‎violation, and unintentional violation are more common in cases, indicating that risky driving behaviors cause more accident and injuries in drivers. Based on this study and previous research, ADHD drivers differed from healthy drivers on a number of indicators of driving performance, specifically, average speed, traveling distance over the speed limit, vehicle control (driving with no hands, driving with the wrong gear), changing lanes/overtaking safely on the motorway, reactions to sudden events, and the expression of frustration or anger with other road users. Barkley et al (1993) claimed that the risk of crashes in drivers with ADHD was 3 to 4 times higher than in other drivers. Other studies that have rejected the above claim but have shown that drivers who have ADHD disorder along with other disorders, such as ODD (oppositional defiant disorder) or conduct disorder (CD), have a higher risk of car accidents than drivers who have only ADHD (30). 

 The ADHD group also had more driving offences and accidents and scored higher on the lapses and violations based on subscales of the Manchester Driving Behavior Questionnaire. In addition, ADHD drivers made negative comments (anger and swearing) significantly more frequently than healthy drivers (10, 11, 27-32). 

The results of this study showed that ADHD and high-risk behavior were more prevalent in the case group who had a traffic accident. Karsazi investigated the role of adult ADHD disorder in high-risk driving behaviors with difficulty mediating emotion regulation. The results indicated that slips and driving errors are directly affected by ADHD symptoms due to their cognitive origins. However, driving disorders, because they are influenced by emotional and motivational factors, are indirectly mediated by difficulty in regulating the emotion regulation of ADHD symptoms.

## Limitation

This study had some limitations that must be considered when interpreting the results. One limitation is that the research community only included men. The most important reason for the overwhelming number of male participants in this study was to observe the principle of the gender ratio of drivers. Many traffic injuries are fatal but they were not included in our study. One of the limitations of this study was the use of self-report questionnaires. However, to reduce this limitation, clinical psychologists completed the questionnaires by interviewing the participants.

## Conclusion

Drivers with ADHD and risky driving behaviors have less control over their cars and drive more violently and faster compared to normal drivers. Also, drivers with ADHD and risky driving behaviors have more chance to cause driving accident. Lack of patients' cooperation was one of the limitations of this study. Also, due to inappropriate physical condition, the patients were not able to fill out the questionnaires.
